# Patients’ priorities around drug-resistant tuberculosis treatment: A multi-national qualitative study from Mongolia, South Africa and Georgia

**DOI:** 10.1080/17441692.2023.2234450

**Published:** 2023-01-01

**Authors:** Annabelle South, Parveen Dhesi, Conor D. Tweed, Bazarragchaa Tsogt, Suzanne Staples, Nestani Tukvadze, Gantsetseg Dorj, Sindisiwe Zaca, Ekaterine Sanikidze, Nasanjargal Purev, Hanif Esmail, Rochelle Burgess

**Affiliations:** a MRC Clinical Trials Unit at UCL, UCL, London, UK; b Mongolian TB Coalition, Ulaanbaatar, Mongolia; c THINK: TB and HIV Investigative Network, Durban, South Africa; d TB Clinical Research Unit, National Centre for Tuberculosis and Lung Diseases, Tbilisi, Georgia; e TB Research and Surveillance Department, National Center for Communicable Diseases, Ulaanbaatar, Mongolia; f Davit Tvildiani Medical University, Tbilisi, Georgia; g Institute for Global Health, UCL, London, UK

**Keywords:** Drug-resistant tuberculosis, Treatment experience, Qualitative research

## Abstract

We conducted qualitative research exploring the treatment experience of people with DR-TB. We held nine focus group discussions with 57 adults undergoing/recently completed treatment for DR-TB in Georgia, Mongolia and South Africa. Translated transcripts were analysed using thematic analysis. We identified three higher order themes: (1) Treatment experience and the role of good relationships with healthcare providers: Treatment duration, pill burden and side-effects were challenging aspects of treatment. Side-effects/symptoms that were visible signs of illness were particularly troubling. Good relations with clinical staff helped combat fear and uncertainty regarding treatment. (2) Mental distress and opportunities for wellbeing: The shame, stigma and isolation people experienced as a result of their DR-TB diagnosis was an important cause of mental distress. No longer being infectious enabled people to resume work and socialising. Positive emotions emerged with good treatment outcomes. (3) Fear and worry along the treatment journey: Participants expressed fears about TB: infecting others; whether they would be able to endure treatment; side-effects; health consequences of treatment. Worries mostly disappeared with successful treatment. Alongside measuring side-effects, time to culture conversion and cure rates, future trials of DR-TB treatments should capture how quickly visible symptoms resolve, quality of life measures, and mental health outcomes.

## Introduction

Drug resistant tuberculosis (DR-TB) is a public health crisis, posing a threat to health security through its contribution to antimicrobial resistance worldwide and acting as a driver of the global tuberculosis pandemic (Udwadia & Furin, 2019; World Health Organisation, 2022c). Around half a million people fall ill with DR-TB each year, of whom only one in three access quality care (World Health Organisation, 2022c). Treatment for DR-TB is long and challenging, involving combinations of drugs with high pill burdens and significant toxicity (World Health Organisation, 2020). Outcomes from DR-TB treatment are far from ideal, with an average of 60% of those treated having successful outcomes in treatment programmes (World Health Organisation, 2022a).

However, recent years have seen the emergence of several new treatment regimens for DR-TB that have cure rates of approximately 80% in clinical trials. These include shorter regimens such as the 6-month bedaquiline, pretomanid, linezolid +/- moxifloxacin regimens for DR-TB(Berry et al., 2022; Conradie et al., 2020; Conradie et al., 2022) and other shorter all-oral regimens that avoid some of the more severe side-effects associated with injectable agents(Goodall et al., 2022; World Health Organisation, 2022b). Within the next few years there are likely to be several approved treatment regimens for DR-TB with similar efficacy, and potentially similar safety profiles as measured by the conventional collection of adverse event data (Working Group on New TB Drugs, 2022).

To date, trials of DR-TB regimens have focused on efficacy measures relating to microbiological cure and serious adverse events, which do not fully capture the breadth of patients’ experience of the disease and treatment. Previous research has shown that pulmonary TB negatively affects physical, mental, and psychosocial health-related quality of life domains (Kastien-Hilka et al., 2017), and that functioning in these domains may remain impaired even after microbiological cure is achieved (Kastien-Hilka et al., 2016). By focusing on microbiological cure, trials may miss out on differences between treatment regimens that are important to people treated for TB and comparisons between regimens could incorrectly conclude there are no significant differences. There are a variety of health-related quality of life tools that try to capture patient-centred outcomes that could be relevant to TB, but it is currently unclear which of these measures best captures what is important to people with DR-TB, and could be used as an outcome measure within a randomised controlled trial. These include FACIT-TB (Abdulelah et al., 2015; Dujaili et al., 2015); EQ-5D (Dion et al., 2004; Park et al., 2021; Saleem et al., 2018); SF-36 (Dion et al., 2004; Lins & Carvalho, 2016; Martinez et al., 2000; Mathai et al., 2016; Spencer et al., 2001); SF-12 (Ohrnberger et al., 2020; Soh et al., 2021); SF-6 (Fu et al., 2019); WHOQOL-Bref (Hawthorne et al., 2006); the St George’s Respiratory Questionnaire (Ferrer et al., 2002; Xu et al., 2009), and the MOS-HIV instrument (A. W. Wu et al., 1997, 1991).

A recent qualitative systematic review of patient perspectives of TB and its treatment high-lighted the following as outcomes of importance to patients: improvements in the signs and symptoms of disease; mortality and survival; treatment failure, success and cure; the adverse effects of treatment; and the impact of treatment on the patient’s ability to function in social, physical, developmental, educational or economic terms (L. E. Hoppe, 2016). The review included 22 studies from Africa, Asia, South America, Central America, North America and Europe. However, most of the included studies in this systematic review do not specify whether participants had drug-susceptible or DR-TB, and given the substantially worse outcomes and more challenging treatment for DR-TB, there may be important differences in priorities for patients with DR-TB compared to drug-sensitive TB.

To address this gap, we carried out qualitative research in Georgia, Mongolia and South Africa to explore the aspects of DR-TB disease and treatment that are of the greatest importance to adults being treated for DR-TB. This work is part of a larger project to identify patient-centred outcome measures for use in randomised controlled trials of DR-TB treatments.

## Methods and materials

This was a multi-national qualitative research study exploring participants’ experiences of DR-TB and its treatment. We used a story stem method to structure focus group discussions (FGDs) exploring the DR-TB treatment journey. Story stem methods are seen as a valuable approach in contexts where stigma, exclusion and shame are associated with either the topic of interest, experiences or identity categories of participants (Clarke et al., 2017; Gravett, 2019). The COREQ checklist can be found in Supplementary File 1.

### Study setting

This study took place in three health facility sites in Mongolia, South Africa, and Georgia. These sites were selected due to the high prevalence of DR-TB in their countries, a representation of different DR-TB treatment regimens being used and because of they had an established background in TB research. South Africa is one of ten countries in the world with the highest prevalence of DR-TB, and co-infection with HIV is common in this setting. There are high levels of unemployment and poverty in South Africa. This site has also participated in international TB trials and previously conducted TB-related qualitative research. Mongolia and Georgia are considered ‘high-burden’ settings for DR-TB, although co-infection with HIV is less common. Both of these sites also have experience in TB-related research. The settings have different healthcare systems, with Mongolia’s being predominantly public, while South Africa has a mix of public, private and NGO healthcare provision, and Georgia operates a national health insurance system, with care provided by private medical facilities. Another consideration in the choice of sites was the number of DR-TB patients they treat, giving us a sufficient pool of potentially eligible participants from which to recruit. Details of each study setting can be found in Supplementary File 2.

### Sampling and recruitment

Inclusion criteria: men and women (aged ≥18 years old) who had completed treatment for DR-TB within the last 12 months, or, who were in the continuation phase (or final three months if receiving a six month regimen) of treatment for DR-TB (Nahid et al., 2019), and were culture negative and clinically well enough at the time of participation were recruited to this study.

Exclusion criteria: those displaying current signs or symptoms of active disease (e.g. cough, fevers).

Participants were purposively sampled by staff involved in DR-TB management or clinical trials at each of the study sites to cover a range of DR-TB regimens, sex, and age. Through our different sites we were also able to recruit both HIV-negative and HIV-positive individuals. Once potentially eligible participants were identified by staff involved in DR-TB management or clinical trials, and they had agreed to being approached by the research team, the research team contacted the potential participant, explained and offered a copy of the Patient Information Sheet, and gave practical details about the FGDs.

### Data collection and tools

The data were collected through nine FGDs (three in each country, each with different participants). These discussions took place between May and August 2021, lasting approximately one to two hours each and following a facilitator guide. FGDs were conducted by researchers at participating sites and audio recorded and transcribed verbatim in the native language before being translated into English by local translators. Transcripts were not returned to participants for comment or correction. Further information about the research team can be found in Supplementary File 3.

A facilitator guide was developed by CT ad RAB in collaboration with patient representatives and site researchers. This included three story stems that represented three stages in the treatment experience of patients diagnosed with DR-TB; beginning treatment, during treatment and ending treatment. Follow-up questions were developed to guide exploration of the issues relating to the participants’ DR-TB experience. Training in using the method was provided for facilitators by RAB. The same story stems were translated into local languages in all FGDs enabling a comparison of the experiences explored by the different groups. The story stems can be found in Supplementary File 4

### Data analysis

The data were analysed using Braun and Clarke’s (Braun & Clarke, 2006, 2019) reflexive thematic analysis through an understanding that thematic analysis is a reflexive process where the creation of themes is underpinned by central concepts.

The data analysis was conducted by PD, AS and RAB. Initially we attempted to extract any possible parts of a story creation from the transcripts. However, once it became obvious that not all focus groups had created stories, we carried out a data-driven thematic analysis of the entire data set. Active reading allowed researchers to familiarise themselves with the data and make notes of possible codes. Following this, researchers discussed their initial thoughts and together created coding groups across the countries involved. The transcripts were coded separately in NVIVO 12 by AS and PD. These codes were exported to Microsoft Excel and used to create a coding frame-work. This allowed prominent experiences of DR-TB treatment amongst the different country groups to be explored and compared. Through this, meaningful coding groups were identified across the countries. These meaningful coding groups were presented to and discussed with the FGD facilitators, after which, RAB, PD and AS were able to create and refine the final themes generated from the data. Participant checking did not take place.

### Ethical considerations

This study received ethical approval from UCL REC (19219/002), South Africa (PharmaEthics Ethics committee Ref: 210423988, 25 June 2021), Mongolia (Medical Ethics Monitoring Committee of the MoH of Mongolia N227, 21 June 2021), Georgia (Local Ethics Committee of the National Centre for Tuberculosis and Lung Diseases). Participants gave informed written consent prior to the start of the FGDs.

## Results

### Participant characteristics

There were 57 participants in total. The sociodemographic and disease data for all participants is summarised in Table 1. A summary of the TB drugs participants had been treated with can be found in the Supplementary File 5 (excluding three South African participants for whom this information was not available). Nearly all participants received bedaquiline (93%), levofloxacin (76%) or moxifloxacin (13%), and most received clofazimine (63%) and linezolid (57%). Only 6% received an injectable agent (kanamycin or capreomycin).

## Findings

### Use of story stems

While in many other studies participants generate stories with a complete narrative arch (a beginning, middle and end) (Clarke et al., 2017; Gravett, 2019) in our study the use of stories/characters varied across sites. However, the character within the story stems enabled the exploration of collective experiences related to DR-TB.

Two groups in Georgia did not attempt to construct a story through use of the story stems. The Mongolian groups and the remaining Georgian group used the story stems to create a dialogue through the character. In this way, the character within the story stems became a platform to explore a collective journey and highlight key experiences related to DR-TB.

In South Africa, the groups related each story stem to the experiences of a character, ‘Jabu’. This is a more traditional approach to the collective use of stems in groups (Clarke et al., 2017; Gravett, 2019). However it did not lead to discussion of their own experiences in the way other countries did and a dialogue was created solely through ‘Jabu’. Under the assumption that ‘Jabu’ was the typical person undergoing DR-TB treatment, discussing Jabu’s experiences in relation to each story stem allowed a reflection of the participants as the real people with real experiences.

### Thematic analysis

The thematic analysis generated three higher-order themes (thematic categories) describing the experience of patients in our international sites: (1) the treatment experience and role of good relationships with healthcare providers, (2) mental distress and opportunities for positive wellbeing, and (3) understanding fear and worry along the treatment journey. Each higher-order theme consisted of themes and sub-themes.

### Treatment experience and the role of good relationships with healthcare providers

The treatment experience was largely shaped by participants’ concerns about treatment and the side-effects they experienced. Supportive interactions and trusting relationships with healthcare providers were seen to facilitate a more positive treatment experience.

### Treatment experience

Across the countries, concerns about treatment were primarily related to the length of treatment and the pill burden (amount of pills and effects of pills). These concerns appeared to cause a significant amount of distress for participants.

In my opinion the worst thing about it is its duration, it was really difficult for me. (Georgia FGD2)

Sometimes when [Jabu] looks at [the medication] she asks herself who is going to swallow this big pill and you ask yourself if you will be able to continue drinking it, it really traumatises her. (South Africa FGD3)

In addition, participants in Mongolia and South Africa frequently discussed side-effects such as nausea and vomiting, fatigue and weakness, alongside their coping strategies for this. Importantly, side-effects or symptoms that were visible signs of their illness were particularly troubling for participants, for example changes in skin colour (Mongolia) or weight loss (South Africa).

It’s so clear to notice that you have had TB. Because we become [these] terribly dark-skinned people. (Mongolia FGD2)

These side-effects were often cited as reasons for potential poor adherence to treatment regimens. Therefore, addressing the aforementioned side-effects was noted by participants to have important implications for the development of future therapies.

I wonder if it is possible to make drugs with no side effects? If there is such a treatment, it is not important to reduce the time, but the side effects. The drugs that would at least not make you feel dizzy, nauseous? (Mongolia FGD1)

Conversely, poor adherence could also be the result of improved health or becoming sputum negative as it may lead to questions around why continued treatment is necessary.

I think she must be asking herself now, if there is a need to continue with medication though there were no traces of the virus [*sic*] in her sputum. What is the purpose of continuing [*with taking*] the pills. (South Africa, FGD2)

Factors that motivated participants to continue with their treatment included encouragement from family and support from the clinic. Furthermore, despite their concerns about treatment and the side-effects experienced, participants often displayed a will to survive.

In order not to die, you have to take this [take TB pills] I tell myself. I love my life now. That’s how I took the pills. (Mongolia FGD2)

I am saying Jabu was accepted by the family with his TB and that encouraged him to take treatment well. (South Africa, FGD1)

### Relationships with healthcare professionals and the healthcare system

Positive, supportive relationships with healthcare providers appeared crucial to the participants’ treatment experience and helped improve adherence to medication.

In Georgia, good communication and the setting of expectations from healthcare professionals enabled participants to better understand their condition and perhaps make sense of the side effects that are happening to them. This built trust between healthcare providers and participants, giving the participants hope moving forward in their treatment.

As I got acquainted with my doctor, our relations became so friendly, she was like a family member for me, I trusted and obeyed her prescriptions without suspicion. (Georgia FGD2)

In South Africa relationships with clinical staff were seen as supportive. Participants discussed being acknowledged as high priority by the clinic.

I think Jabu will get support from sisters at the clinic, you get people that helps psychologically, you get counselling, she will get support there. (South Africa, FGD2)

In addition, participants in South Africa discussed the importance of doing what the doctor told them to do, and how accurate information about TB provided at the hospital could help tackle fears about TB, further emphasising the importance of establishing trust between the healthcare services providing TB treatment and the participants.

Nowadays there are people who are in hospitals, who are there to explain to you about the sickness. They tell you to do this and this, by the time you go [to the hospital], [you have] all the information, especially [for]TB. (South Africa FGD3)

In contrast, some participants from Mongolia described a lack of support and information from healthcare professionals that exacerbated negative treatment experiences.

I think doctors and nurses should be respectful and flexible toward patients. It would make patients feel better if they explained the effects and intent of each pill. (Mongolia FGD1)

Comparing the relationships with healthcare providers across the countries demonstrate that where there was good support from healthcare professionals and encouragement from family, participants felt encouraged to continue medication. Where there was no such support, adhering to treatment may have been made more difficult.

In Georgia and South Africa, where the treatment experience and relationships with the healthcare providers appeared positive, patients who had been successfully treated talked of being evidence that DR-TB could be cured, and seemed willing to provide support to others going through treatment.

He will be able to advise other people about TB that it can be cured, he is a living testimony. (South Africa FGD1)

This could help to further facilitate trust and understanding among patients undergoing treatment for DR-TB.

### Mental distress and opportunities for positive wellbeing

The diagnosis and treatment of DR-TB was associated with mental distress for many participants, shaped by the extent to which their illness damaged social relationships. These relationships were influenced at both societal and interpersonal levels.

### Mental distress is shaped by the fracture of relationships due to the DR-TB diagnosis

Stigma surrounding TB, particularly as an infectious disease, appeared to lead to participants being excluded from social circles, including family, friendship groups, neighbours and work colleagues. Some participants described being ridiculed by their friends, leaving them feeling disappointed and alone.

They will discriminate, they will say I should sleep alone, not with them, use my own dishes not use dishes that is used by everyone. (South Africa FGD2)

My friends made fun of this and laughed at me for it. You got bone tuberculosis. This is disgusting said to me [by] my friends. (Mongolia FGD2)

This socially imposed isolation (through interpersonal verbal discrimination and the anticipation of stigmatisation) led to a loss of social support networks and created an isolating experience for participants.

MDR is scary because it will be like this is how we live now, that happen to other people, how will they say and how will they look at you? Will she receive the same love as before? (South Africa FGD3)

Participants from Mongolia and South Africa talked about the personal mental distress experienced as a result of the DR-TB diagnosis. In Mongolia, some participants blamed themselves for becoming infected with TB. Mental distress was a prominent concern in the Mongolian and South African data and included feelings of depression and suicidal ideation, loss of confidence, unhappiness and feeling low.

Then I immediately felt very low. I wanted to die instead of having those anguishes. (Mongolia FGD3)

He won’t have confidence like before, like before having these diseases, he will always have that thing at the back of his mind that he was once diagnosed with TB. (South Africa FGD1)

In these countries, TB was less readily accepted as a curable disease compared to Georgia. In Mongolia, participants’ initial perceptions of TB were extremely pessimistic with conflicting perceptions about whether TB was curable.

I got depressed, I didn’t think I would recover, I thought I’d die now, felt similar to having a cancer. (Mongolia FGD2)

In South Africa, despite some awareness of TB, the underlying perceptions of DR-TB were largely negative and the information available to them could be overwhelming.

Most people, if you talk about TB, they think you are going to die. Lot of people think TB is fatal. (South Africa FGD3)

In Georgia, a diagnosis does not appear to fracture the social world as significantly as it does within participants from Mongolia and South Africa. As previously described, in Georgia interactions with healthcare professionals created trust and facilitated a supportive environment. This is coupled with the perception that TB was not a particularly stigmatising disease meant reactions to diagnosis were not overwhelmingly of fear and shame.

[My family’s] reaction also was normal, I think such attitude is part of our Georgian culture, we don’t expel so easily a person from the society. (Georgia FGD1)

Relationship dynamics discussed by participants in Georgia demonstrated that supportive relationships with family and friends and non-discriminating behaviour was a motivating factor for participants undergoing DR-TB treatment. This allowed for the maintenance of healthy social relationships.

My family helped me psychologically, the fact that I had people next to me that worried and cared about me affected me positively. (Georgia FGD3)

In this way, participants from Georgia appeared to have a better pre-existing capacity for positive wellbeing. They appeared to experience less discrimination and more support from family and friends, perhaps because of lower levels of societal stigma, compared to those from Mongolia and South Africa.

### Changes due to DR-TB

The fracture of the social world experienced in Mongolia and South Africa was exacerbated by the changes participants experienced due to their TB diagnosis and treatment experience. Many were unable to carry out their usual activities of daily living due to the physical health implications of DR-TB.

Oh, [I] felt so tired, often panted, climbing to 1–5 stairs of the building became difficult and tiring. It is as if I lost all my strength. (Mongolia FGD1)

In addition, participants described being unable to work. This was often due either to their poor physical health or discrimination by employers leading to participants losing their jobs.

Yes, fired me. I got disappointed, I was jobless, how am I going to support my children? I thought. What to do? (Mongolia FGD2)

I am an electric welder, but this profession is forbidden to me, so I lost my job. (Georgia FGD2)

This reduced capacity for employment was a major concern in the focus groups, and had important financial implications for people living with TB. For example, participants had difficulty buying food and accessing healthcare facilities, further complicating their treatment.

Some people will not take pills because they will say they did not have food therefore they could not take so [many] pills in an empty stomach. (South Africa FGD2)

Lack transport money to go to the clinic if the clinic is too far from him. (South Africa FGD1)

The inability to carry out normal activities of daily living, confounded by increased financial hardship was a source of further mental distress for the participants.

### Enabling the return to normal: opportunities for positive wellbeing emerge with the reduction in symptoms and side-effects

Positive emotions amongst participants only emerged with potential success of treatment. This was strongly linked to a reduction of symptoms and side-effects that allowed participants to start returning to normal. This was demonstrated most clearly within the South African focus group discussions.

Indicators of successful treatment (e.g. negative sputum culture) led to the participants being able to discuss positive emotions such as hope, optimism, thankfulness and happiness.

I think that she was thankful that she listened to the doctors when telling her to take treatment, with her good doing today she is receiving such news. (South Africa FGD2)

Participants considered resolution of visible symptoms as a return to normal. Persistence of visible symptoms was perceived to hinder their ability to return to normal, whether due to their personal physical health or others reactions/ perceptions of their recovery (discrimination and stigmatisation). Weight gain and increased appetite were seen as signs of recovery.

She is gaining weight that makes her to be confident that she will also go back to work she work she will not be discriminated, they will see that she is being treated. (South Africa FGD3)

As soon as I went back to work, I became the best employee within a week. (Mongolia FGD 1)

Feeling better allowed them to resume household chores, allowing a feeling of returning to normal.

Things like moving furniture at home and also taking the broom and sweeping the house, he would be encouraged because he is now able to do those things. (South Africa FGD1)

Therefore, as participants’ physical manifestations of disease and the limitations this imposed disappeared and their treatment comes to an end (perceived cure) they are able to rebuild their social connections.

She will now have hope that she can be loved by all those who distance themselves even friends and family they will love her. (South Africa FGD2)

I wanted to go back to work when I’m all better. Now I’m working; healthy as a dog. I’m also loving the fact that I’m gaining weight. The most important factor for getting healthy is to take your pills consistently. It’s great when you are fully recovered. (Mongolia FGD2)

### Understanding fear and worry along the treatment journey

Fear and worry were prevailing feelings during the discussions throughout the countries. Participants talked about their fear and worry in relation to their DR-TB diagnosis, its treatment, and the implications of DR-TB and its treatment on their relationships and their future.

### Fear and worry about DR-TB and the treatment experience

The majority of participants expressed a fearful reaction to their diagnosis.

At this moment I am thinking: what shall I do now? Or what awaits me in future? What will happen to me? (Georgia FGD1)

Notably, fear relating to the infectious nature of DR-TB was prominent across all of the countries. This included fear of how they had acquired DR-TB, fear of spreading DR-TB to loved ones and fear of being reinfected.

When I got to know I was infective, my first reaction was that – may I have infected anybody? […] if had infected someone, I would have felt guilty. (Georgia FGD2)

I do fear that I might get infected again. I fear that I might get infected from someone or some other places. (Mongolia FGD2)

In South Africa participants raised fears about how far the disease had gone for them to require such long treatment.

I think she must be asking herself that as she will be treated for 8–12 months while most of the time it usually takes up to 6 months, she must be asking herself on how far the infection have gone? (South Africa, FGD2)

Fear and worry about DR-TB treatment focussed on the length, side-effects and success of treatment. The length of treatment and pill burden of DR-TB seemed intimidating for some participants, leading them to worry about whether they will be able to adhere to treatment.

Telling someone, who is under a lot of psychological distress, to just simply take that many pills is scary. (Mongolia FGD1)

He is wondering if he will be able to take the treatment accordingly for all these months required from him. (South Africa FGD1)

Uncertainty about the side-effects they may experience was a worry for some participants before commencing treatment. As treatment progressed through the second story stem participants discussed specific side-effects that may be particularly concerning, such as changes in skin colour, leg pain, neuropsychiatric side-effects and vomiting.

Some people scare you by saying you will go crazy sometimes, see things that are not seen. (South Africa FGD2)

There were also worries that treatment may not be successful or that TB may return.

Since she is still having side effect, they might be scared that the TB might come back. (South Africa FGD2)

He is scared thinking what if the treatment he will receive does not work or is he going to die. (South Africa FGD1)

Participants in South Africa had worries that the treatment may cause other diseases.

Is there any other diseases that might come from taking this treatment? (South Africa FGD1)

However, fear of the health consequences of stopping treatment may have helped motivate participants to continue taking their medication.

But he is also scared that if he stops taking medication because of side effects that might develop, he is scared about what might happen if he stops taking medication. (South Africa FGD1)

### Fear and worry about the impact of DR-TB on relationships and the future

Participants worried about the longer-term implications for their futures, whether they would go back to being their old selves, and how this experience would impact their relationships with others.

In South Africa and Mongolia participants worried about how their illness would affect their relationships, with concerns about rejection, discrimination and isolation.

Firstly, what scared us most if we get such news is to what to tell our partners. (South Africa FGD2)

In Georgia, participants felt they had to choose between financial security and health, ultimately participants often came to the conclusion that they had to undertake treatment.

I thought about it, because I had such a job, that I had to work in any case. I need money for my family. Then I decided that in this condition I couldn’t deal with job. (Georgia FGD1)

Uncertainty around what will happen in the future, how future plans may be affected, and what happens after they are cured were a source of worry. Participants worried about whether or not they will ever go back to being the person they were before their illness.

He is scared if he will ever go back to the Jabu that he was, to his normal state. (South Africa FGD1)

Despite some fears that the disease may come back or have longer-term health effects, very few participants talked about fears or worries in relation to the final story stem, suggesting fear and worry reduced as treatment showed signs of success (e.g. culture conversion).

## Discussion

### Summary of key results

We found that, across three very different settings, people’s experience of DR-TB goes beyond the physical symptoms of disease and side-effects of treatment. When embarking on treatment for DR-TB, people have serious concerns about the treatment duration, pill burden and side-effects. Support and clear communication from healthcare providers can help to alleviate these concerns, but does not always take place. In this project, we found that a diagnosis of DR-TB is often associated with significant mental distress, often caused by the fracture of social relationships due to stigma around the disease, fear of infecting close family members, fear for their future, inability to carry out activities of daily living and the economic consequences of this. As treatment shows signs of success, through reduction in visible symptoms of the disease and then culture conversion, the process of returning to normal can begin. Participants talked about many fears and worries along their treatment journey, about their health and whether they would recover; their treatment and its long-term impact; and the impact of the disease on their relationships with other, and future life. As treatment succeeds, these fears and worries recede.

### Our findings in context

Our study adds to the understanding of the experience of people with DR-TB through the multicountry nature allowing us to draw tentative comparisons between the settings; for example, the seeming absence of stigma around TB in Georgia compared to Mongolia and South Africa meant Georgian focus group participants experienced less of a fracture of their social world, and associated challenges. Carrying out this research in just one country would not have allowed us to capture this insight. All comparisons between the three countries in this study can only be tentative, as Table 1 shows there were several demographic differences between participants in the three countries, which could account for some of our observed differences.

It has been well documented that some drugs used to treat TB (such as cycloserine, fluoroquinolones and ethambutol) are associated with psychological side-effects (S. Wu et al., 2016). Our findings suggest that mental distress is not always due to drug side-effects, but also the (often sub-stantial) negative impact of DR-TB diagnosis and treatment on social and economic well-being, which should be considered when interpreting results around mental distress from clinical studies of DR-TB treatment.

Our findings are consistent with previous research carried out with people with MDR-TB in Thailand, which found that all participants experienced emotional difficulties when first diagnosed, including fear, stigma, confusion and sadness (Numpong et al., 2022). Research in central India among people with MDR/XDR-TB found that the high pill burden, treatment duration, side-effects and economic impact of the disease all posed challenges for treatment adherence (Nigam et al., 2021). Similarly, in Brazil people treated for MDR-TB have reported challenges with adherence due to the side-effects of treatment (Santos et al., 2021). Research among people treated for DR-TB in Eswatini found that allowing treatment to take place at home, rather than as inpatients at a health facility, positively influenced quality of life through allowing some continuity of everyday life and relationships, but fear of the being infected was a concern among family members (Burtscher et al., 2021).

Our finding around the importance of good information and clear communication about DR-TB and treatment for alleviating concerns about treatment reinforces previous findings from research among people with DR-TB and HIV in South Africa (Daftary et al., 2021). They also found challenges around stigmatisation, with families and social networks avoiding, disrespecting and reducing support to people with DR-TB (Daftary et al., 2021), similar to some of the experiences reported by participants in our study. Poor mental health has been associated with the experience of fractures within a patient’s social world – which consists of social relationships, structures, and systems that are linked to their survival (Burgess & Campbell, 2014; Summerfield, 2012). This seemed particularly pertinent for participants from Mongolia and South Africa, who experienced stigma and isolation due to the disease.

While we found that fears and worries receded as treatment was completed, previous research around the post-treatment health of people with rifampicin-resistant tuberculosis in South Africa has found that returning to normal was not straightforward, with all participants reporting physical, social, psychological and economic changes as consequences of the diagnosis and treatment (Loveday et al., 2021). Some of our participants reported not returning to their previous occupation due to inability to cope with the physical demands, or desire to protect themselves from reinfection. Similarly, some participants talked about the restoration of social and family relationships, but not all relationships were restored.

### Implications of our research

Our research has a number of implications for DR-TB treatment programmes. The challenges of living with side-effects of DR-TB treatment came up repeatedly in our focus groups. Our findings have been communicated to the National TB Programme in Mongolia, which used the findings to inform training of TB clinicians around the management of side-effects, and planning of side-effect medications from the government funds. The mental health aspect of the experience of DR-TB treatment, and the need for support with this, was a prominent theme in the data. The Mongolian National TB Programme has established the socio-psychological support team. Updated National TB guidelines of 2021 have also included options for TB patients taking their medications monitored online by health and community health care workers.

Our work will help to also help to inform decisions around what patient-centred outcome measures are used in trials of DR-TB treatment in order to address the issues that matter to people with DR-TB. It has already informed the choice of quality-of-life measures used in the Unite4TB adaptive phase II trial platform protocol.

### Strengths

Three researchers were involved in the FGD analysis enhancing researcher triangulation. In addition, the meaningful coding groups were presented to and discussed with the FGD facilitators. Through this, the facilitators were able to discuss their thoughts and experiences of leading the FGDs, helping to achieve good reflexivity throughout the process. In addition, it enabled facilitators to provide feedback on the accuracy and relevance of the meaningful coding groups, enhancing the study’s credibility.

Our study is the first to use story completion methods to gather data in the context of DR-TB. Story completion methodology is particularly relevant to qualitative research investigating DR-TB, a condition that can be heavily stigmatised. Using story stems in the context of data collection allowed access to a wide range of responses, importantly socially undesirable ones (Clarke et al., 2017; Clarke et al., 2019). In addition, it suits sensitive topics because it does not necessarily require personal revelations of experience. In this way it can reduce social desirability bias; this can be a particular issue when discussions involve clinicians and peers. In attempting construction of the story, the participants anchored to the character in a way that allowed them to elaborate on their own experiences. In addition to occasionally mapping the story of the character, the participants related their own experiences to the story mapped out by the stems provided. Therefore, despite being unable to create stories as anticipated in the study design, we obtained rich qualitative data exploring participants’ experiences of DR-TB through the use of story stems in the FGDs. While we were initially concerned that participants would be unwilling to share their own experiences because of the sensitive nature of the topic, we found that participants were very keen to discuss their experience, giving them the opportunity for their voices to be heard.

This study is one of the first qualitative studies to explore the treatment experiences of people with DR-TB being treated predominantly with all-oral bedaquiline-based regimens, which have different side-effect profiles to older regimens with injectable drugs. As national and international guidelines have now increasingly moved to recommend all-oral bedaquiline-based regimens (World Health Organisation, 2022b), our research is timely and relevant for many countries.

The study took place over three international sites in different continents; Mongolia, South Africa and Georgia. This geographic spread allowed capture of patient perspective across a wide range of socio-economic demographics, cultures, ethnicities and HIV co-infection rates. Most previous qualitative studies in the area of DR-TB have been carried out in single countries.

### Limitations

DR-TB is a global public health crisis (World Health Organisation, 2022c), while our study was limited to three countries. Therefore, it is inevitable that some experiences of DR-TB have not been captured due to limited geographical involvement. Comparisons between the different countries in this study need to be interpreted carefully, with awareness that there are multiple demographic and contextual differences between the focus group participants in these countries, including the treatment regimens used. This limits our ability to draw conclusions about differences between the three countries. Because our analysis was data driven, themes were only created when the concept or experience was common across all locations. What varied was the emphasis on particular aspects of that experience across countries. As such, we do not emphasise the comparisons across countries, as they are so rooted to the contextual differences in each location. Some participants may have received treatment as part of a clinical trial, meaning their experiences may not be typical of people treated under national TB programmes.

## Conclusion

Further work is ongoing to explore patient priorities around the different outcomes highlighted in this research and identify how these priorities map onto existing patient-centred outcome measures currently used in TB trials, to inform the design of future trials of DR-TB regimens.

DR-TB diagnosis and treatment affects people’s physical and mental health, as well as their social relationships and economic wellbeing. Trials of new DR-TB treatment regimens need to look beyond microbiological outcomes to adequately capture the outcomes that matter to people treated for DR-TB. Alongside microbiological cure trials need to consider time to resolution of visible symptoms and side-effects, which often lead to stigma; mental health and other psychosocial outcomes. Many people affected by DR-TB are economically active and have dependents, so the impact of different treatment regimens on their ability to be economically active and carry out their caring responsibilities should be an important consideration when evaluating treatment options.

## Supplementary Material

COREQ checklist

Supplementary file 2: Study settings

Supplementary File 3: Research team and reflexivity

Supplementary File 4: Story Stems

Supplementary File 5: Drug Treatment Data

## Figures and Tables

**Table 1:** Participant characteristics.

Participant characteristics		Georgia *N* = 22			Mongolia *N* =15			South Africa *N* = 20			TOTAL *N* = 57	
		N	%		N	%		N	%		N	%
Sex	Male	15	68%		5	33%		11	55%		31	54%
	Female	7	32%		10	67%		9	45%		26	46%
Participant Highest Qualification	No formal education	0	0%		1	7%		1	5%		2	4%
	Primary school (grade 1–7)	1	5%		3	20%		3	15%		7	12%
	Secondary school (grade 8–11)	11	50%		5	33%		11	55%		27	47%
	Tertiary (diploma/ degree)	10	45%		6	40%		5	25%		21	37%
Currently Employed	Yes	16	73%		8	53%		4	20%		28	49%
	No	6	27%		7	47%		16	80%		29	51%
Participant relationship status	Single	10	45%		3	20%		15	75%		28	49%
	Married living with partner	9	41%		8	53%		3	15%		20	35%
	Married not living with partner	1	4%		3	20%		1	5%		5	9%
	Unmarried living with a life partner	0	0%		1	7%		1	5%		2	4%
	Divorced	1	5%		0	0%		0	0%		1	2%
	Other	1	5%		0	0%		0	0%		1	2%
TB treatment status	On treatment	0	0%		12	80%		6	30%		18	32%
	Completed treatment	22	100%		3	20%		14	70%		39	68%
Significant Past Medical History^1^	Yes	2	9%		5	33%		17	85%		24	42%
	No	20	91%		10	67%		3	15%		33	58%
Most recent TB diagnosis	Drug-susceptible	0	0%		0	0%		0	0%		0	0%
	Rifampicin-resistant	0	0%		6	40%		0	0%		6	11%
	Multidrug-resistant	22	100%		9	60%		20	100		51	89%
	Pre-extensively drug-resistant	0	0%		0	0		0	0%		0	0%
	Extensively drug-resistant	0	0%		0	0		0	0%		0	0%
Number of times participant has been treated for any TB disease	0	22	100%		7	47%		1	5%		30	53%
	1	0	0%		8	53%		7	35%		15	26%
	2	0	0%		0	0%		9	45%		9	16%
	≥3	0	0%		0	0%		3	15%		3	5%
Previous treatment interruption	Yes	0	0%		1	7%		4	20%		5	9%
	No	22	100%		14	93%		16	80%		52	91%
Complications & Side-Effects^2^	Yes	15	68%		15	100%		18	90%		48	84%
	No	7	32%		0	0%		2	10%		9	16%
Site of most recent disease	Pulmonary	22	100%		13	87%		20	100%		55	96%
	Other	0	0%		2	13%		0	0%		2	4%
HIV status	HIV-negative	22	100%		15	100%		5	25%		42	74%
	HIV-positive	0	0%		0	0%		15	75%		15	26%
Age	Age range	19–67			30–65			26–60				
	Mean age	37			39			41				

1 HIV, Asthma, ischaemic heart disease, Hypertension, Diabetes

2 2Nausea, Liver dysfunction, Neuropathy, Arthalgia, Rash, Other reported.

## Data Availability

The anonymised data that support the findings of this study are available from the corresponding author, AS, upon reasonable request.
