# Supplementary material for: Patients’ priorities around drug-resistant tuberculosis treatment: A multi-national qualitative study from Mongolia, South Africa and Georgia
Source: Glob Public Health. Author manuscript; Available in PMC 2024 Jul 31. (PMC7616316; doi:10.1080/17441692.2023.2234450)
Supplement: Supplementary file 2: Study settings [file EMS197758-supplement-Supplementary_file_2__Study_settings.docx]

**Supplementary file 2: Study settings**

**National Centre for Communicable Disease, Ulaanbaatar, Mongolia**

Mongolia is one of the TB burden countries in the Western Pacific Region and in 2021 it was included in the WHO list of 30 high MDR TB burden countries. In 2020, the WHO estimated there were 3,861 new TB cases, of which 179 (4.6%) were MDR-TB cases in Mongolia. The treatment success rate among new bacteriologically positive TB cases has remained at 85% over the past five years. In contrast, the treatment success rate among MDR/RR-TB cases has declined from its peak of 67.5% in 2016 to 56.4% in 2019, mostly attributed to increasing loss to follow up (LTFU). The National TB Patient Cost Survey 2018 showed that on average about 70% of TB affected households faced catastrophic costs due to TB, with a higher proportion for the DR-TB affected households (85%) (World Health Organisation, 2020a). The healthcare system in Mongolia is predominantly public, with TB and other infectious disease care provided free of charge. Nationwide, specialised TB care services are provided by the National Centre for Communicable Diseases (NCCD), district and province TB dispensaries, as well as Prison Hospital, National Center for Mental Health and Enerel hospital for homeless people. The NCCD is the main hub for prevention, diagnosis, treatment, research and surveillance of infectious diseases (NCCD, 2022). It treats approximately 50% of cases of TB each year, of which around 10% have DR-TB. The study was conducted by researchers in Ulaanbaatar, the capital city of Mongolia, in collaboration with the Mongolian TB Coalition (MTC) and NCCD.

**TB and HIV Investigative Network (THINK), Durban, South Africa**

In South Africa, there were an estimated 191,074 new TB cases in 2020, of which 6,784 (3.6%) were MDR-TB cases. In the same time frame, the treatment success rate among new bacteriologically positive TB cases in South Africa was estimated at 79%. However, this fell to 65% for MDR-TB. The total TB case fatality ratio (estimated mortality/ estimated incidence, 2020) was 19% in South Africa. South Africa has both a public and a private healthcare system (World Health Organisation, 2020b). However, national and international non-Governmental Organisations (NGOs) also play a big role in health system capacity, particularly surrounding communicable diseases, and child and maternal health. In South Africa, this study was conducted by the TB and HIV Investigative Network (THINK), a public health delivery and research organisation, across two sites, Hillcrest and Pietermaritzburg in the KwaZulu-Natal province (THINK, 2022). Through their clinical trial work they treat approximately 50-150 patients per project depending on the design. Through their programme activities they are involved, directly or indirectly, in the care of approximately 9500 DSTB cases and 400 DRTB cases per year.

**National Centre for Tuberculosis and Lung Disease, Tbilisi, Georgia**

There were 1,671 new TB cases estimated by the WHO in Georgia in 2020, 202 (12.1%) of which were MDR-TB cases. Treatment success rate among new bacteriologically positive TB cases in Georgia for 2020 was estimated at 85% but this fell to 67% for MDR-TB (World Health Organisation, 2020c). The National Centre for Tuberculosis and Lung Disease (NCTLD) in Tbilisi has in-patient and out-patient TB facilities, with a 97-bed inpatient unit dedicated to treatment of patients with DRTB (NCTLD, 2022). In 2022 they treated approximately 354 cases of TB, of which around 71 were DR-TB. The NCTLD reports to the Georgian Ministry of Health and was the primary study site in Georgia.

**References**

National Center for TB and Lung Disease. (2022). Available at: <https://www.vumc.org/vecd/national-center-tb-and-lung-disease-n>

National Centre for Communicable Disease. (2022). Available at: <https://www.nccd.gov.mn/>

THINK. (2022). Available at: <https://www.think.org.za/>

WHO. (2020a). Tuberculosis profile: Mongolia. Available at: <https://worldhealthorg.shinyapps.io/tb_profiles/?_inputs_&entity_type=%22country%22&lan=%22EN%22&iso2=%22MN%22>

WHO. (2020b). Tuberculosis profile: South Africa. Available at: <https://worldhealthorg.shinyapps.io/tb_profiles/?_inputs_&entity_type=%22country%22&lan=%22EN%22&iso2=%22ZA%22>

WHO. (2020c). Tuberculosis profile: Georgia. Available at: <https://worldhealthorg.shinyapps.io/tb_profiles/?_inputs_&entity_type=%22country%22&lan=%22EN%22&iso2=%22GE%22>
