# Supplementary material for: Patients’ priorities around drug-resistant tuberculosis treatment: A multi-national qualitative study from Mongolia, South Africa and Georgia
Source: Glob Public Health. Author manuscript; Available in PMC 2024 Jul 31. (PMC7616316; doi:10.1080/17441692.2023.2234450)
Supplement: Supplementary File 3: Research team and reflexivity [file EMS197758-supplement-Supplementary_File_3__Research_team_and_reflexivity.docx]

# Supplementary File 3: Research team and reflexivity

|  | **Georgia** | **Mongolia** | **South Africa** |
| --- | --- | --- | --- |
| **Focus group facilitator(s)** | E. Sanikidze | Bazarragchaa Tsogt (BTs)  Gantsetseg Dorj (GD), Nasanjargal Purev (NP) | Nonhlanhla Ndondo (NN)  Sindisiwe Zaca (SZ)  Siyabonga Myeni (SM) |
| **Credentials of the facilitator(s)** | MD, PhD | BTs works as research fellow in TB for the last decade. GD and NP are working with the NTP in charge of TB surveillance and research | NN - Bachelor of Science Honors in Psychology 2014  SM - Masters in Development Studies obtained in 2020  SZ- Bachelor of psychology degree obtained in 2009. |
| **Occupation of the facilitator(s)** | Study coordinator, Advocacy, communication and social mobilization specialist | GD and NP-NTP epidemiologists; BTs- Medical doctor, researcher | Research Assistants |
| **Gender of the facilitator(s)** | Female | All 3 female | NN – Female  SZ – Female  SM – Male |
| **Experience and training of the facilitator(s)** | Experienced in interviewing | BTs, GN and NP has extensive research experience, mainly in quantitative research. However, qualitative training on “Story stem” FGDs conducted by the MRC CTU researchers twice by online. BTs, the local PI, organized two more training for the team members on the study protocol, how to undertake FGDs and ethics of inviting the participants and obtaining the consents. Role playing or mock FGDs within the team using the “story stems” were important to understand the study details. | NN – Over 7 years' experience in public health research  Bachelor of Science Honors in Psychology  SM – 5 years research experience  Masters in Development Studies obtained in 2020  SZ- Bachelor of psychology degree obtained in 2009. 12 years' experience in data collection for clinical and social sciences research projects. |
| **Was a relationship established between facilitator and participants prior to study commencement?** | No | A team of six people worked in this study from Mongolia. Two members, from the authors, GD, has contacted the participants by phone together with one other team member, who did not included in the list of authors. | During participant recruitment, rapport was established while educating the participant about the study and attending to participants’ concerns and questions that they had before deciding to participate in the study. Prior to the study, the participants knew the facilitator from the clinic where they collect their TB treatment. |
| **What did the participants know about the researcher?** | That she is a TB doctor, ethics and law and Advocacy, communication and social mobilization specialist, experienced in communication and interviewing | Participants know the name and work place of the researcher who contacted them. On the day of the FGD, team members who were present there all were introduced. | The participants knew the facilitators as research assistants who works at the health facility that the participants use to obtain their TB treatment. |
| **Facilitator characteristics** | TB doctor, researcher, ethics and law and Advocacy, communication and social mobilization specialist | All facilitators work in TB, but not directly involved in the diagnosis and treatment of TB patients. All were interested what were the DR TB patients’ experience and what important for them overcoming such difficult disease; mainly to listen to them and to find ways to improve care and services, as these were the first FGDs undertaken in Mongolia from DR TB patients. Two facilitators shared the roles during the FGD to capture all important points said by the participants. | The facilitator(s) understood the research objective. They had the ability to make everyone comfortable, encouraged everyone to speak up, encouraged a respectful tone while diligently managing the pace of the discussion.  The facilitators were new to the story stems approach and were particularly concerned as to whether the approach would work practically in their setting, but after training they were more comfortable.  There was no bias and assumptions other than a genuine interest in understanding the aspects of treatment, such as side effects, that were ‘important’ to patients from the patients’ point of view. |
| **Method of approach** | Face to face interview in the focus group, maintaining autonomy, respect of dignity and don’t harm principles, creating friendly atmosphere | In 2021, 108 DR TB patients were registered. From this cohort, the team has selected those who meet the Exclusion and Inclusion criteria. One of the main criteria for us were patients living in Ulaanbaatar, where the study site is located. 34 patients were invited and 15 were part of the FGDs.  We tried to include participants who have completed their treatment within 12 months and those who were on treatment.  All participants contacted by phone calls and those who were on treatment, but were hospitalized at the DR TB wards of the NCCD, were given the Patient information sheet with Consent form prior to FGDs a week ago. Assigned, trained nurse have taken the written Informed consent for those in the hospital on the day of the FGD.  Only those who consented and met the Inclusion criteria were invited to be part of the study. Those who completed treatment have given the Consent on the day of the FGD. | Some participants were approached face to face when they came for their clinic visits. Others were contacted telephonically. Closer to the day of the focus group, phone calls were made to remind the participants of the meeting. |
| **Was anyone else present besides the participants and researchers?** | Assistant person | 1-2 team members were present to assist in notetaking and logistics. | A social worker was present during some of the focus group discussions. A second research assistant (mentioned above) to assist with note taking. |
| **Were field notes made during and/or after the inter view or focus group?** | Yes | Notes were taken during the focus group discussions. | The team made field notes during the focus group discussions. Notes were taken using Flip charts, which were also visible to the participants and also note pads were used to take notes for future referencing and reporting purposes. |
