# Supplementary material for: Patients’ priorities around drug-resistant tuberculosis treatment: A multi-national qualitative study from Mongolia, South Africa and Georgia
Source: Glob Public Health. Author manuscript; Available in PMC 2024 Jul 31. (PMC7616316; doi:10.1080/17441692.2023.2234450)
Supplement: Supplementary File 4: Story Stems [file EMS197758-supplement-Supplementary_File_4__Story_Stems.docx]

**Supplementary File 4: Story Stems**

**Story Stem 1: Beginning of Treatment**

[Patient] has been told they have drug-resistant tuberculosis after being unwell for several months. The doctor meets with them and their partner to explain that treatment will last for at least nine to 12 months, and that treatment will likely have side effects. The doctor explains the disease is curable but the treatment will need to be taken daily to achieve this and reduce infectiousness. After the appointment [patient] is quiet and their partner asks what is on their mind.

**Story Stem 2: During Treatment**

After completing 4 months of treatment, [patient] attends for a clinic review and is told that the treatment has been working because there are no tuberculosis bacteria in their sputum and their weight is improving. The doctors say that they need to perform some tests to make sure there are no dangerous side effects from the treatment. There will be least 5-8 more months of treatment. [Patient] has been trying to make sure they take the medication every day but this hasn’t been easy. After the appointment their partner turns to [patient] and comments that maybe life can go back towards normal now and [patient] could get back to work.

**Story Stem 3: End of Treatment**

[Patient] is nearing the end of treatment. At their last visit, the doctor told them the treatment has worked and their TB should be cured. They will need to attend for a final clinic review when they will have a chest X-ray done and be assessed. [Patient] is talking with their partner about the treatment and their plans for the future.
