# Supplementary material for: Patients’ priorities around drug-resistant tuberculosis treatment: A multi-national qualitative study from Mongolia, South Africa and Georgia
Source: Glob Public Health. Author manuscript; Available in PMC 2024 Jul 31. (PMC7616316; doi:10.1080/17441692.2023.2234450)
Supplement: Supplementary File 5: Drug Treatment Data [file EMS197758-supplement-Supplementary_File_5__Drug_Treatment_Data.docx]

**Supplementary File 5: Drug Treatment Data**

| Drug Name | Number of Participants receiving named drug | | |  |
| --- | --- | --- | --- | --- |
|  | Mongolia (n=15) | Georgia (n=22) | South Africa (n=17) | Percentage receiving drug |
| Bedaquiline | 11 | 22 | 17 | 92.6% |
| Capreomycin (injectable) | 1 | 0 | 0 | 1.9% |
| Clofazimine | 11 | 7 | 16 | 63.0% |
| Cycloserine | 5 | 7 | 0 | 22.2% |
| Delamanid | 1 | 0 | 0 | 1.9% |
| Ethambutol | 12 | 0 | 10 | 40.7% |
| Isoniazid | 7 | 0 | 9 | 26.9% |
| Kanamycin  (injectable) | 2 | 0 | 0 | 3.7% |
| Levofloxacin | 15 | 11 | 15 | 75.9% |
| Linezolid | 2 | 12 | 17 | 57.4% |
| Moxifloxacin | 0 | 6 | 1 | 13.0% |
| Pretomanid | 0 | 15 | 1 | 29.6% |
| Prethionamide | 9 | 0 | 0 | 16.7% |
| Pyrazinamide | 15 | 6 | 5 | 48.1% |
| Terizidone | 0 | 0 | 5 | 9.3% |

| Country | Regimen | No. of participants |
| --- | --- | --- |
| Mongolia | Bedaquiline, Clofazimine, , Ethambutol, Levofloxacin, Prothionamide, Pyrazinamide | 6 |
|  | Bedaquiline, Clofazimine, Ethambutol, Isoniazid, Levofloxacin, Prothionamide, Pyrazinamide | 3 |
|  | Bedaquiline, Clofazimine, Cycloserine, Levofloxacin, Linezolid | 1 |
|  | Ethambutol, Levofloxacin, Kanamycin, Pyrazinamide, Isoniazid | 1 |
|  | Cycloserine, Isoniazid, Levofloxacin, Kanamycin, Pyrazinamide | 1 |
|  | Cycloserine, Ethambutol, Isoniazid, Levofloxacin, Pyrazinamide | 1 |
|  | Capreomycin, Cycloserine, Ethambutol, Isoniazid, Levofloxacin, Pyrazinamide | 1 |
|  | Bedaquiline, Clofazimine, Cycloserine, Delamanid Levofloxacin, Linezolid | 1 |
| Georgia | Bedaquiline, Pretomanid, Linezolid | 9 |
|  | Bedaquiline, Pretomanid, Moxifloxacin, Pyrazinamide | 6 |
|  | Bedaquiline, Levofloxacin, Linezolid, Clofazimine, Cycloserine | 7 |
| South Africa | Bedaquiline, Clofazimine, Levofloxacin, Linezolid, Terizidone | 4 |
|  | Bedaquiline, Clofazimine, Ethambutol Levofloxacin, Linezolid | 1 |
|  | Bedaquiline, Clofazimine, Ethambutol, Isoniazid Levofloxacin, Linezolid | 5 |
|  | Bedaquiline, Clofazimine, Levofloxacin, Linezolid, Pyrazinamide | 1 |
|  | Bedaquiline, Clofazimine, Ethambutol, Isoniazid Levofloxacin, Linezolid, Pyrazinamide | 4 |
|  | Bedaquiline, Linezolid, Moxifloxacin, Pretomanid | 1 |
|  | Bedaquiline, Clofazimine, Linezolid, Terizidone | 1 |
